# Effects of Magnetic Resonance Imaging With Axial Traction of the Thumb Carpometacarpal Joint on Articular Cartilage Visibility: A Feasibility Study

**DOI:** 10.7759/cureus.22421

**Published:** 2022-02-21

**Authors:** Akira Ikumi, Sho Kohyama, Shun Okuwaki, Masaki Tatsumura, Yuki Hara, Takeo Mammoto, Takeshi Ogawa, Yuichi Yoshii, Haruo Kawamura, Masashi Yamazaki

**Affiliations:** 1 Department of Orthopedic Surgery and Sports Medicine, Tsukuba University Hospital Mito Clinical Education and Training Center, Ibaraki, JPN; 2 Department of Orthopedic Surgery, Kenpoku Medical Center Takahagi Kyodo Hospital, Ibaraki, JPN; 3 Department of Orthopedic Surgery, Kikkoman General Hospital, Chiba, JPN; 4 Department of Orthopedic Surgery, Faculty of Medicine, University of Tsukuba, Ibaraki, JPN; 5 Department of Orthopedic Surgery, National Hospital Organization, Mito Medical Center, Ibaraki, JPN; 6 Department of Orthopedic Surgery, Tokyo Medical University Ibaraki Medical Center, Ibaraki, JPN

**Keywords:** joint space width, articular cartilage visibility, thumb carpometacarpal joint, magnetic resonance imaging, axial traction

## Abstract

Objectives

The objective of this study was to verify the usefulness of magnetic resonance imaging (MRI) with axial traction of the thumb for observing articular cartilage.

Materials and methods

Eleven healthy adult volunteers (39.7 ± 7.4 years) without thumb carpometacarpal joint arthritis or trauma were included in this study. A 3-tesla (3T) MRI (Magnetom Skyra, Siemens Healthineers AG, Munich, Germany) of the right thumb with axial traction applied by a finger trap with three traction weights (0, 2, and 5 kg) was performed. A 3D T2* multiecho data imaging combination (MEDIC) was selected to visualize the articular cartilage. After multiplanar reconstruction, sagittal and coronal images of the thumb carpometacarpal joint were used to evaluate the articular cartilage visibility and joint space widths at five locations. Articular cartilage visibility was evaluated using our original classification method that used the percentage of the cartilage detectable area. The Friedman test was used to compare the differences between each traction weight and location.

Results

Articular cartilage visibility significantly improved with axial traction. The average joint space widths with the 5-kg application were 1.9 ± 0.8, 3.9 ± 0.6, 2.0 ± 0.9, 3.9 ± 1.1, and 2.5 ± 1.4 mm at the center, volar edge, dorsal edge, radial edge, and ulnar edge, respectively. The joint space widths significantly increased proportionally with the traction weight at all locations. The joint space widths at the volar and radial edges were significantly greater than those at other locations.

Conclusion

Applying axial traction to the thumb increased the joint space widths and improved the visibility of the articular cartilage in the carpometacarpal joint on MRI.

## Introduction

The thumb carpometacarpal (CMC) joint is a saddle joint that connects the first metacarpal bone and the trapezium and can be moved in multiple different directions during daily activities such as pinching or grasping due to its anatomical characteristics [1,2]. The incidence of thumb CMC joint arthritis is high, occurring in >15% of adults aged >30 years and one-third of postmenopausal women, despite it being a non-weight-bearing joint [3-6]. Thumb CMC joint arthritis is usually diagnosed on the basis of the patient’s history, physical examination, and radiographs. The Eaton and Littler classification system is widely used to determine the staging and severity of such types of arthritis [7,8]. However, the degree of damage in the thumb CMC joint cartilage is not accurately assessed because the Eaton and Littler classification is based only on radiographs. A study reported that the intra- and inter-examiner reliabilities of this classification are low [9]; other studies have reported a poor correlation with clinical symptoms and intraoperative articular cartilage findings [10,11].

Magnetic resonance imaging (MRI) is widely used to evaluate the damage to articular cartilage. However, accurate evaluation of the thumb CMC joint cartilage is difficult due to its anatomical complexity and relatively small size compared with large joints such as the hip and knee. Although some reports have evaluated the articular cartilage of the thumb CMC joint using MRI [12-14], an accurate method of evaluation has not yet been established because of the overlap of the articular cartilage between the first metacarpal bone and the trapezium as well as the underestimation of the degree of cartilage damage compared with other pathological findings [15]. Therefore, to enhance the visibility of the articular cartilage, we have attempted to perform MRI while applying axial traction to the joint. This method of applying axial traction to improve visualization of articular cartilage has been previously used to observe other joints, including for osteochondritis dissecans in the capitellum [16].

This study aimed to examine the effects of MRI with axial traction of the thumb CMC joint on the visibility of articular cartilage among healthy volunteers.

## Materials and methods

Study population

This study was approved by the institutional review board (IRB) of the first author's hospital (no. R2-06) and was performed following the ethical standards laid down in the 1964 Declaration of Helsinki and its later amendments or comparable ethical standards. We enrolled 11 healthy volunteers without a history of a thumb injury and symptoms. All volunteers were hospital staff members. The first author provided detailed information about the study (after IRB approval) within the hospital premises and recruited the volunteers. Informed consent was obtained from each volunteer after thoroughly explaining the objective, method, and any expected complications of the study.

Image acquisition

MRI was performed at the Kenpoku Medical Center Takahagi Kyodo Hospital between January 2021 and March 2021. We used a 3-tesla (3T) whole-body MRI system (Magnetom Skyra, Siemens Healthineers AG, Munich, Germany) using a four-channel 3T special purpose coil (Siemens Healthineers AG, Munich, Germany). For the MRI sequence, a three-dimensional T2* multiecho data imaging combination (MEDIC) scan was used with the following parameters: slice thickness, 0.2 mm; slice gap, 0.15 mm; field of vision, 130 mm × 130 mm × 78 mm; matrix, 384 × 292; time to repeat, 20 ms; echo time, 11.0 ms; and flip angle, 25°. The required time according to the protocol was 5 min and 43 s for each imaging. The volunteers were asked to lay supine on the table with their arms outstretched and forearms in pronation at the side of the body. The hand under observation was centered parallel to the long axis of the gantry.

Application of axial traction during MRI

**Figure 1:**
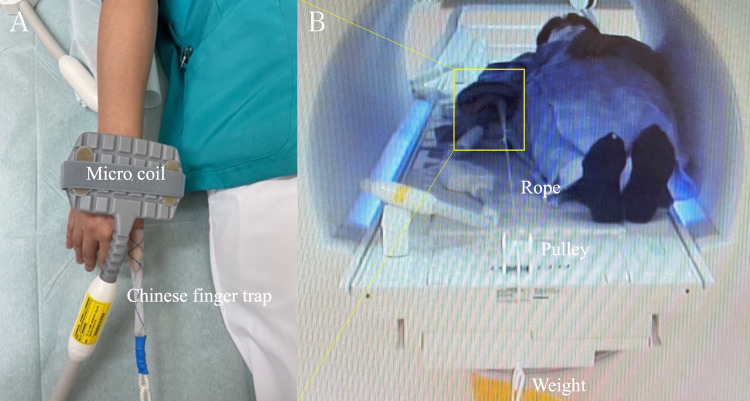
Application of axial traction during thumb carpometacarpal joint MRI (A) The volunteer’s thumb was enclosed within a Chinese finger trap and pulled distally. (B) The Chinese finger trap was connected to the traction weight using a nonelastic rope routed through the pulley system.

MRI was initially performed without traction, followed by MRI with traction. After performing the initial MRI without traction, the volunteer’s thumb was enclosed within a Chinese finger trap (Allen® Sterile Mesh Finger Traps, AliMed, Inc., Massachusetts, USA) using a rope. The other end of the rope was hung over the edge of the MR table via a pulley system and attached to nonmagnetic traction weights (Figure 1).

The ideal traction weight for the thumb has not been determined to date; therefore, traction weights of 2 and 5 kgs were used based on previous literature [17].

Image analysis

We evaluated the following items: the effects of traction on the joint space width, articular cartilage outline visibility, and pain and discomfort during imaging with traction. In this study, joint space width was defined as the space between opposing articular cartilages within the target joint. All MR images were independently evaluated by two orthopedic surgeons (with 13 and six years of clinical experience, respectively). All study images were interpreted on a workstation (Materialise Mimics, version 20.0; Materialise, Belgium); this workstation was also used to obtain multiplanar reconstructed (MPR) images. Specifically, coronal and sagittal images were reconstructed such that they were parallel to the longitudinal axis of the first metacarpal bone. This procedure was performed by the first author for all images. The images were initially enlarged, and the gray-scale contrast was adjusted to optimize the visualization of the structure being assessed. The images were then randomly numbered to minimize bias among examiners.

Measurement of the joint space width

**Figure 2:**
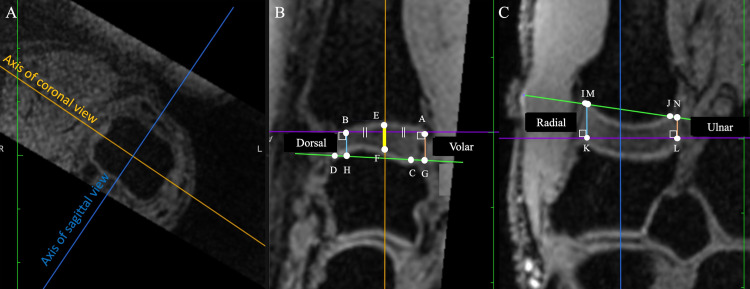
Definition of measurement points (A) The coronal and sagittal axes were defined using the axial plane at the first metacarpal bone head. (B) Sagittal image of first carpometacarpal joint. Distances E-F, A-G, and B-H were defined as the center, volar, and dorsal distances, respectively. (C) Coronal image at the first carpometacarpal joint. Distances K-M and L-N were defined as radial and ulnar, respectively.

The joint space width was measured on the sagittal and coronal images at the center of the proximal articular surface of the first metacarpal bone. On the sagittal image, the AB line, the line through both the volar (point A) and dorsal (point B) borders at the proximal articular surface of the first metacarpal, and the CD line, the line through both volar (point C) and dorsal (point D) borders of the distal articular surface of trapezium, were drawn at first. Then, a perpendicular line was then drawn at the center of the AB line, and the intersection points of the articular surface of the first metacarpal were labeled as point E and the intersection points of the articular surface of the trapezium were labeled as point F. Furthermore, the intersection points of the perpendicular line drawn from point A and B to the CD line were labeled as points G and H. On the sagittal image, the IJ line, the line through the radial (point I) and ulnar (point J) borders of the proximal articular surface of the first metacarpal, and the KL line, the line through the radial (point K) and ulnar (point L) borders at the distal articular surface of the trapezium, were drawn first. Subsequently, the intersection points of the perpendicular line drawn from points K and L to the IJ line were defined as points M and N. The distance between points E and F was defined as the center of the joint space width, that of points A-G and B-H was defined as volar and dorsal of the joint space width, and that of points I-M and J-N was defined as radial and ulnar of the joint space width (Figure 2).

Assessment of the articular cartilage outline visibility

**Figure 3:**
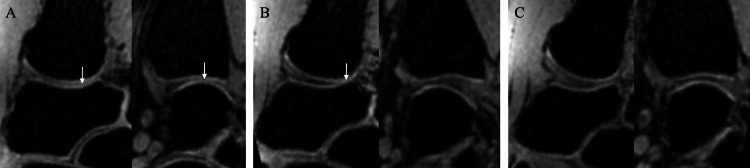
Articular cartilage outline visibility grade (A) Grade 0 (poor): The articular cartilage outline was visible in <50% of the entire range when facing the opposing articular cartilage. (B) Grade 1 (intermediate): ≥50% but <100% of the articular cartilage outline was clearly visible in the range when facing the opposing articular cartilage. (C) Grade 2 (complete): 100% of the articular cartilage outline was clearly visible in the entire range when facing the opposing articular cartilage. The contact area of each articular cartilage is indicated by the white arrows.

Articular cartilage outline visibility was graded using the following three-point scale on each sagittal and coronal image in which the joint space width was measured: 2 (complete), when 100% of the articular cartilage outline was clearly visible in the entire range when facing the opposing articular cartilage; 1 (intermediate), when ≥50% but <100% of the articular cartilage outline was clearly visible in the range when facing the opposing articular cartilage; and 0 (poor), when the articular cartilage outline was visible in <50% of the entire range when facing the opposing articular cartilage (Figure 3).

Assessment of pain and discomfort during MRI with axial traction

The visual analog scale (VAS) was in a questionnaire format to assess the pain and discomfort during MRI with axial traction and was completed by each volunteer immediately after undergoing MRI with axial traction (0, minimum; 100, maximum).

Statistical analyses

We used GraphPad Prism 8 (GraphPad Software, LLC., CA, USA) for all statistical analyses. All data were tested for normal distribution using the Shapiro-Wilk test. Only the VAS score followed the normal distribution. We used a one-way analysis of variance (ANOVA) to assess the differences in the VAS scores for each traction weight. None of the data on articular cartilage outline visibility and joint space width followed normal distribution due to the small sample size. Therefore, the Friedman test was used to assess the differences in the joint space widths and articular cartilage outline visibility between each traction weight. A P-value < 0.05 was considered significant.

## Results

In this study, six of the 11 volunteers were men and five were women, with a mean age of 39.4 ± 7.4 (range, 27-49) years. Demographic data of the volunteers are presented in Table 1.

**Table 1:** Demographic data of the volunteers CMC: Carpometacarpal.

No.	Sex	Age (Years)	History of thumb CMC joint injury	Symptoms of thumb CMC joint arthritis
1	Male	37	None	None
2	Male	49	None	None
3	Female	45	None	None
4	Female	43	None	None
5	Male	29	None	None
6	Male	30	None	None
7	Male	40	None	None
8	Female	41	None	None
9	Male	27	None	None
10	Female	49	None	None
11	Female	43	None	None

**Figure 4:**
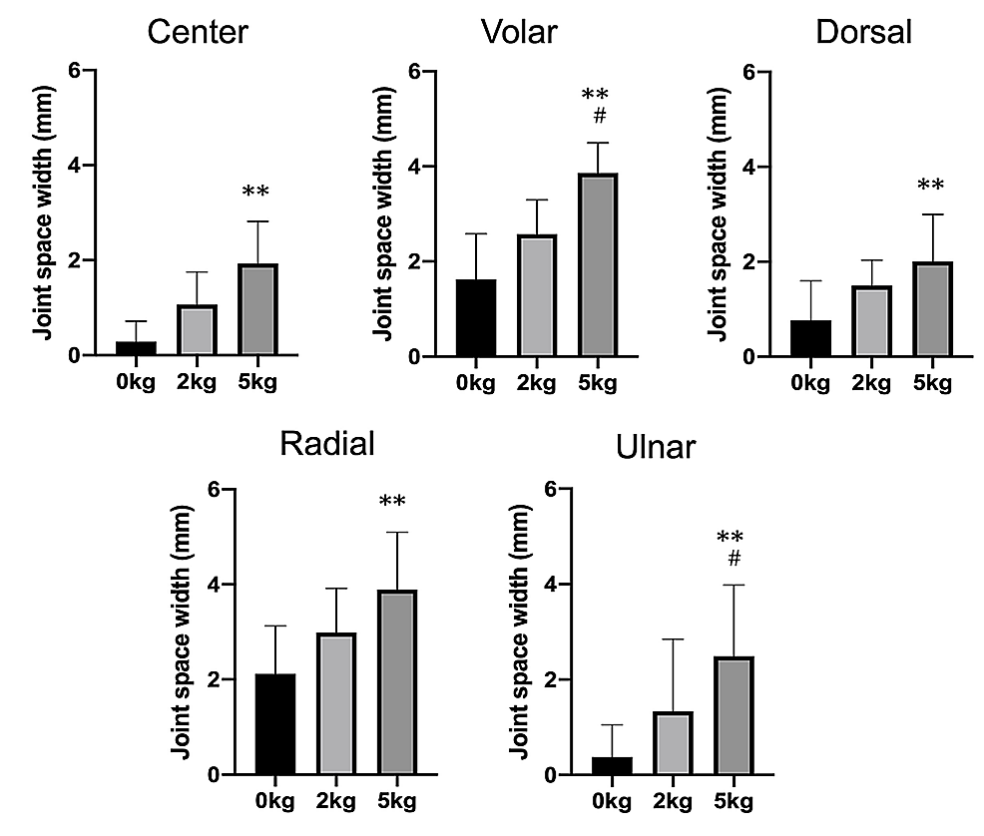
Joint space widths at each point (median ± standard deviation) Joint space widths gradually widened after axial traction. At all points, significant differences were observed between the 0-kg and 5-kg tractions. **P < 0.01 compared with 0-kg traction. ^#^P < 0.05 compared with 2-kg traction.

The joint space width significantly increased at all points with the 5-kg traction than with no traction. No significant differences in the joint space widths were observed between the 2-kg traction and non-traction at all points (Figure 4).

**Figure 5:**
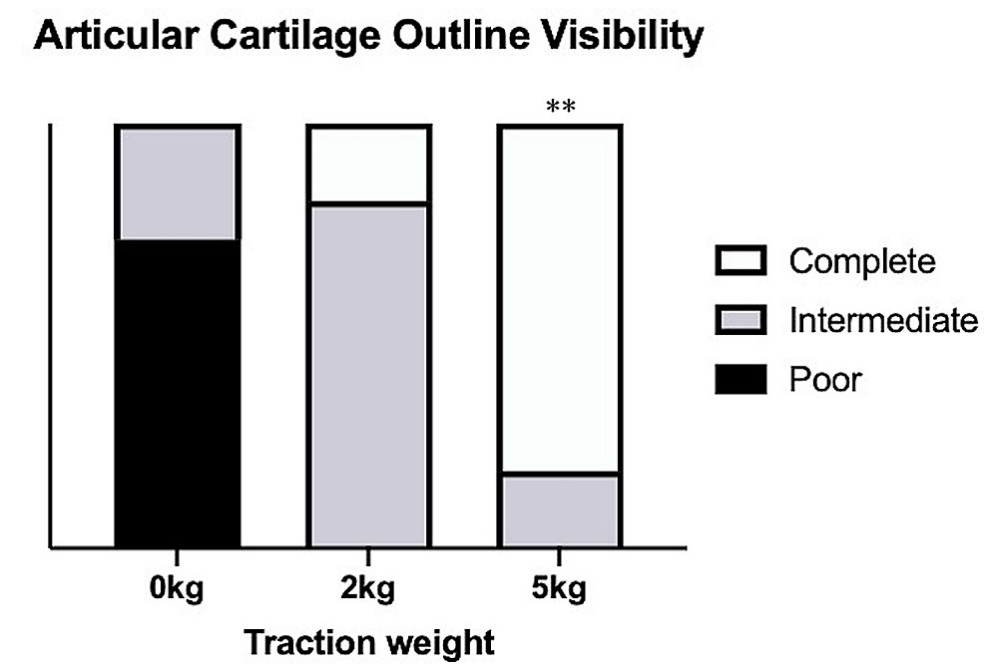
Visibility of the articular cartilage outlines **P < 0.01 compared with the 0-kg traction.

On comparing each point, the volar and radial points were found to have significantly wider joint space width for all traction weights than the center, dorsal, and ulnar. The visibility of the articular cartilage outlines significantly improved with the 5-kg traction (P < 0.01), with approximately 82% of volunteers showing complete visibility of the articular cartilage with the 5-kg traction (Figure 5).

**Figure 6:**
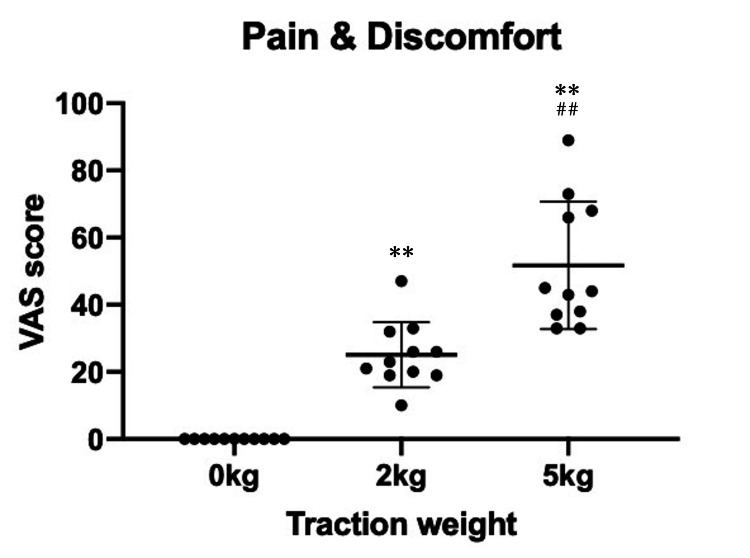
Visual analog scale scores of the pain and discomfort during traction VAS: Visual analog scale. **P < 0.01 compared with the 0-kg traction. ^##^P < 0.01 compared with the 2-kg traction.

The mean VAS score for pain and discomfort during MRI with axial traction was 0 (0-0) in non-traction, 25 (10-47) in 2-kg traction, and 52 (33-89) in 5-kg traction (Figure 6).

The scores significantly increased with traction (P < 0.01); however, the pain and discomfort were resolved within 15 min after completing the traction and removing the finger trap in all volunteers.

## Discussion

Our results showed that the cartilage outline visibility of the thumb CMC joint on MRI was significantly better when axial traction was applied to the thumb.

Traction of the thumb

Joint traction during diagnostic testing and treatment has already been applied in multiple situations [18,19]. Guntern et al. recently demonstrated that a 3-kg distraction load on the wrist significantly expands the radiocarpal and midcarpal joints, particularly the radioscaphoid and lunocapitate spaces [18]. Lee et al. reported that traction significantly improves the cartilage surface visibility for standard MRI of the wrist, although the effect was not as obvious as that seen with MR arthrography or MR arthrography with traction [17]. Orthopedic surgeons have also used axial traction of the wrist for treatment and diagnosis. The distraction of the wrist by axial traction helps realign the fragments after displacement fractures of the distal radius. Axial traction has been used as a carpal stress test in the diagnosis of scapholunate ligament injuries in radiology [20]. Furthermore, four previous studies have reported the advantages of applying axial traction during MR arthrography: one at the shoulder [21], two at the hip [22,23], and one at the knee [24]. Although the articular cartilage visibility significantly improved by increasing the traction weight, the pain and discomfort caused by traction were also significantly increased in this study. An increase in the traction weight results in increased tightness of the finger being tested. We suspected that the pain during traction was caused by vascular insufficiency due to the use of the Chinese finger trap. The maximal traction weight in this study (5 kg) is acceptable in a clinical setting because 4.5 kg (10 pounds) of traction is usually applied for fingers when performing wrist or thumb arthroscopy [17,19,25]. However, the traction of the thumb in patients with thumb CMC joint arthritis may exacerbate pain and other symptoms, although these symptoms disappear within a short period in healthy volunteers. Further research is warranted to determine the optimal traction weight for the thumb.

MRI sequences to observe the articular cartilage

MEDIC was used to evaluate the articular cartilage in this study. Its T2*-weighted gradient-echo sequence is specifically designed for musculoskeletal and neuroradiological purposes and combines up to six echoes in a single image, leading to a higher signal-to-noise ratio (SNR) and reduced susceptibility [26-28]. A 3D-MEDIC is useful in the diagnosis of fibrocartilaginous and ligamentous pathologies due to its high intrinsic SNR and high resolution of the 3D data stack [26,27]. Compared to other 3D sequences of the wrist, 3D-MEDIC exhibits a high contrast and SNR as well as the best visibility of fibrocartilaginous and ligamentous tissues [26]. In this study, articular cartilage is defined as a white line contacting the subchondral bone. Therefore, our results suggest that the MEDIC sequence can describe articular cartilage in the same way that it can be used to describe fibrocartilaginous and ligamentous tissues. Therefore, the optimal sequence to describe the articular cartilage in MRI should be examined in a study that compares several MRI sequences in the future.

Importance of articular cartilage evaluation for the thumb CMC joint

Various surgical techniques have been previously reported for thumb CMC joint arthritis [29]. For patients with mild osteoarthritic changes (Eaton classification stage 1 or 2), ligamentoplasty, arthroscopic synovectomy, and first metacarpal osteotomy are selected for thumb CMC joint preservation surgery, whereas, for patients with advanced osteoarthritic changes (Eaton classification stage 3 or 4), arthroplasty such as ligament reconstruction and tendon interposition, arthrodesis, and artificial joint replacement is generally selected as the thumb CMC joint non-preservation surgery. Although the clinical outcome is generally good with each type of treatment [30], Ogawa et al. have reported symptomatic improvement with joint preservation surgery even in patients with advanced osteoarthritic changes [25]. We believe that joint preservation surgery is preferable for younger patients or those who are involved in heavy labor, although a risk of osteoarthritic change progression exists even after a long-term joint preservation surgery.

Badia et al. reported a treatment algorithm based on intraoperative thumb CMC joint arthroscopic findings in 2006 [11]. Depending on the degree of articular cartilage damage or loss, joint preservation or non-preservation surgery is selected in this algorithm. This algorithm is more innovative than the Eaton classification as it assesses the articular cartilage damage and loss with higher accuracy before deciding the surgical technique. However, several limitations, such as the inability to preoperatively evaluate the articular cartilage, invasiveness, and the need to decide the surgical technique intraoperatively, were encountered. Our results demonstrated that the axial traction MRI of the thumb CMC joint could be used to preoperatively evaluate the articular cartilage condition, which will allow the selection of the optimal surgical technique that reflects the articular cartilage condition rather than depending on the Eaton classification.

This study has several limitations. First, the number of study participants was small. Second, we did not examine the use of MRI with axial traction for patients with thumb CMC joint arthritis. Third, the axial traction system in this study could not control the rotation force for the thumb CMC joint. A slight twist caused by axial traction may affect the measurement of joint space widths at each point. Therefore, adding a system to control the rotation force during axial traction is necessary in future studies. Finally, we did not validate the concordance rate between MRI findings and intraoperative or pathological findings. To address these problems, the usefulness of this method should be verified in patients with thumb CMC joint arthritis, and the concordance rate must be verified with intraoperative and pathological findings in the future.

## Conclusions

Axial traction of the thumb increased the joint space widths and improved the visibility of articular cartilage in the thumb CMC joint on MRI. Our results suggest that axial traction MRI can be used to evaluate the articular cartilage in a noninvasive manner in patients with thumb CMC joint arthritis, and it can be used to obtain useful information that will help to select the optimal surgical procedure.
